# Case of Chorea, or Dance of St. Vitus, Cured by the Carbonate of Iron

**Published:** 1824-09

**Authors:** C. F. Vandeburgh

**Affiliations:** Liverpool.


### ART. VII.—*Case of Chorea, or Dance of St. Vitus, cured by the Carbonate of Iron.* By C. F. VANDEBURGH, M.D. Liverpool.

ANN LINDON, an interesting girl of fourteen years of age, was seized (agreeably to her mother's description, while engaged in her usual occupation of selling vegetables in the New Market,) with convulsive motions in the left leg and arm, to a degree that prevented her from walking. She dragged the affected leg after her: neither could she lift her hand to her mouth, without frequent attempts before she succeeded. She has tried numerous medicines and nostrums for upwards of three weeks, without benefit, but gradually became worse.

On the 1st of April, she consulted me. I found her labouring under confirmed chorea, distortion of the muscles of the face, violent convulsive motions of the head and extremities, with total inability to walk, or bring any thing to the mouth; the eyes were in constant motion; attended with an involuntary discharge of urine, likewise of saliva from the mouth, with loss of articulation; the abdomen prominent; bowels costive; pulse natural; appetite good. The involuntary motions continue during sleep; the mind was also considerably impaired.

Practitioners differ greatly in the treatment of this disease, some being advocates for electricity, others for depletion; and nearly the whole range of the Pharmacopœia has been exhausted even, in some instances, without success. But it is not my intention to tire my readers with a lengthened detail of the various methods of practice already adopted. I endeavoured to conquer the disease by purgatives, so judiciously recommended by

Dr. James Hamilton, and ordered the following mixture—℞. Magnesiæ Sulphat. ℥j. Infusi Rosæ ℥ij. Infusi Sennæ ℥iij. fiat mistura, cujus cochlearia tria magna quarta quaque hora sumantur, with the intention of cleansing the primæ viæ of their acrid contents, and continued it for eight successive days. The patient had seldom less than four or five evacuations daily, with no other success than removing the prominency of the abdomen, and rendering the fæces perfectly natural.

The 9th April, I commenced giving half a drachm of ferri subcarbonas, four times a-day, in molasses; and, if the bowels were costive, a small wine-glassful of the cathartic medicine before mentioned.

On the 17th April, the convulsive motions of the lower extremities were less frequent; intellects much improved. Ordered the subcarbonate of iron to be increased to two scruples four times a-day, and the patient to be immersed in the river every morning; which, notwithstanding the coldness of the atmosphere, she appeared to enjoy.

On the 24th April, I perceived great amendment: the involuntary motion had subsided during sleep; the eyes and features became more natural; articulation improved; and, without help, she felt able to walk a short distance. The ferri subcarbonas and sea-bathing were continued.

The 4th May, the power of speech was perfectly regained; the involuntary motions nearly subdued. The same treatment was pursued, accompanied with as much exercise as possible; and, on the 12th May, had the satisfaction of seeing her perfectly restored, and able to attend to her former occupations.

*Liverpool; 15th May,* 1824.

P.S.—Agreeably to Mr. B. Hutchinson's, of Southwell, mode of treatment, (to whom great merit is due,) I have also the pleasure of communicating, that I have given the ferri subcarbonas, in large doses, in two cases of tic douloureux. One, Mr. Potter, of Liverpool, who had suffered upwards of two years, (the complaint was perfectly removed in the space of a fortnight;) the other, Mr. Samuel, of the same place, who had only suffered a short time.

